# Microstructure of Additively Manufactured SUS316L Stainless Steel with SrO Heterogeneous Nucleation Site Particles

**DOI:** 10.3390/ma18215061

**Published:** 2025-11-06

**Authors:** Yoshimi Watanabe, Shimon Sekiyama, Mami Mihara-Narita, Tomokazu Moritani, Hisashi Sato, Kaname Fujii, Ayahito Saikai, Masato Ono

**Affiliations:** 1 Department of Physical Science and Engineering, Nagoya Institute of Technology, Nagoya 466-8555, Japannarita.mami@nitech.ac.jp (M.M.-N.); moritani@nitech.ac.jp (T.M.); sato.hisashi@nitech.ac.jp (H.S.); 2 Industrial Research Institute of Ishikawa, Kanazawa 920-8203, Japan; 3 Industrial Research Center of Shiga Prefecture, Ritto 520-3004, Japan

**Keywords:** additive manufacturing (AM), directed energy deposition (DED), heterogeneous nucleation site, SUS316L stainless steel, SrO

## Abstract

It is known that the addition of SrO heterogeneous nucleation site particles can refine the microstructure of SUS316L stainless steel additively manufactured (AMed) by powder bed fusion (PBF). In this study, this idea was confirmed by directed energy deposition (DED). However, there are several types of DED machines, and the energy system and the material supply system of these machines are different depending on each machine. In this study, the grain refinement behavior and the formability of AMed SUS316L stainless steel with the addition of SrO heterogeneous nucleation site particles are evaluated using a single-beam type LAMDA 200 machine and a multi-beam type ALPION Series machine. The size of the melt pool made by the ALPION Series machine is smaller than that of the LAMDA 200 machine, which results in a shorter residence time in the liquid state of the melt pool for the ALPION Series machine. The grains formed in the inoculated sample manufactured by the ALPION Series machine under the unidirectional scanning strategy are found to be refined compared to those in the uninoculated sample. On the other hand, it is found that the formation of defects and the crystallographic texture observed in the samples manufactured by the LAMDA 200 machine is suppressed by the addition of SrO heterogeneous nucleation site particles. These differences between the ALPION Series and LAMDA 200 machines would come from the differences in the melting state, including temperature, cooling conditions, and re-heating.

## 1. Introduction

**Figure 1:**
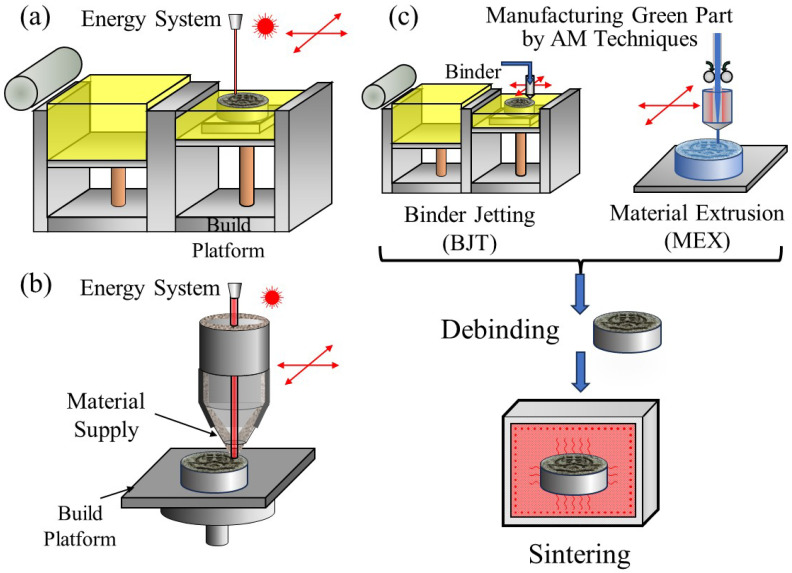
Schematic illustrations of the most common AM methods for metals: (**a**) PBF, (**b**) DED, and (**c**) application of powder metallurgy. The red double ended arrows indicate the movement of the energy system, deposition head and print head.

Recently, additive manufacturing (AM) has provided new pathways and opportunities for the metal material manufacturing field. In the AM process, parts are manufactured layer by layer using a 3D digital model based on the principle of discrete stacking [1]. A wide variety of AM processes have been proposed to create a 3D part. The three most common AM processes for metals are the powder bed fusion (PBF) method, the directed energy deposition (DED) method, and the process based on sintering, including the binder jetting (BJT) method [2]. Schematic illustrations of the most common AM methods for metals are shown in Figure 1. Figure 1a shows a schematic illustration of the PBF method. In this method, some areas are selectively melted by the focused energy, either an electron beam or a laser beam, after a thin layer of powder is spread. The selective melting is repeated layer by layer to build a final component. The re-melting of previous layers during the melting of the current layer allows the current layer to adhere to the rest of the part [2]. Figure 1b shows a schematic illustration of the DED method. During the DED method, the energy focused by a laser beam, electron beam, or arc generates a melt pool into which the feedstock is deposited. In this method, the feedstock is either powder or wire. The third method is the process based on sintering, which is analogous to the metal injection molding (MIM) process. The only difference in the process based on sintering compared to MIM is the shaping technology used to manufacture a green part. Green parts can be manufactured by BJT, material extrusion (MEX), and so on, which are then debinded and sintered to obtain a fully metallic part. A schematic illustration of the sintering-based process is shown in Figure 1c. Among these processes, the DED technique has recently drawn significant attention due to its potential applications in various areas of material processing ranging, from coatings, prototyping, and the repair of high-value components, to the manufacturing of precision-dimensional controlled complex objects [3].

**Figure 2:**
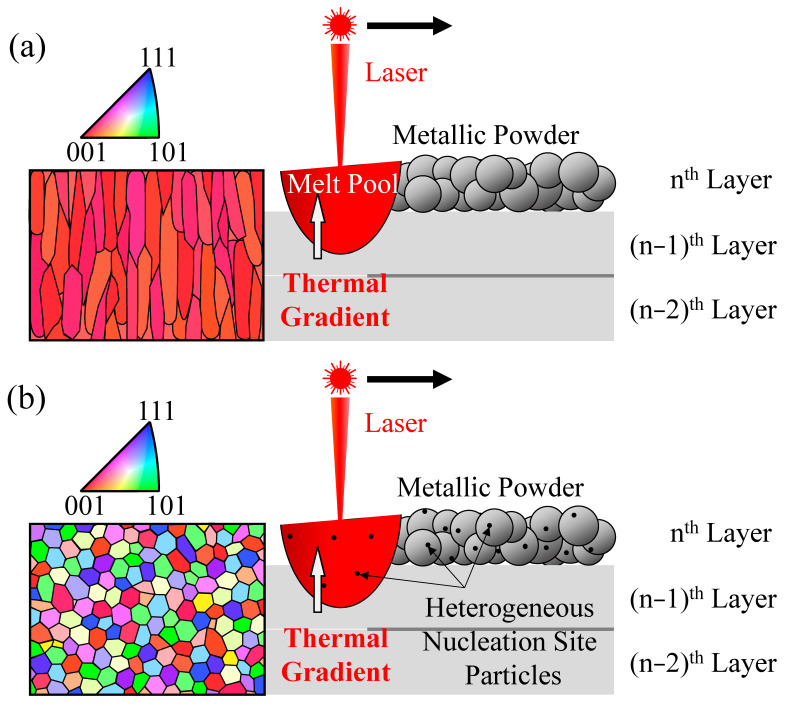
Microstructural evolution during the PBF method using (**a**) conventional metallic powder and (**b**) proposed powder with heterogeneous nucleation site particles. The color map at the left side presents crystal plane orientation observed from building direction.

In AM using PBF and DED methods, which involve the melting and solidification of metals, the temperature gradient in the building direction is large [4]. Since the solidification front moves along this direction, a crystallographic texture is formed. A schematic illustration of the formation of the microstructure of AM samples is shown in Figure 2. Since the preferred direction of crystal growth for cubic metals is <001> [5], <001> texture forms along the building direction of AM samples using conventional metallic powder (Figure 2a). If this phenomenon is considered positively, it can be used to control the microstructure of the AMed sample [6]. However, there are negative aspects when AM is used to manufacture structural parts. When a liquid phase transforms into a solid phase, a solid–liquid interface is formed, which requires supercooling. However, in actual solidification of metals, solidification occurs with a small amount of supercooling due to the presence of a heterogeneous nucleation site [7]. This phenomenon is used in industrial applications by inoculating a grain refiner containing heterogeneous nucleation site particles [8]. This phenomenon can also be applied to AM [9,10,11,12,13]. Figure 2b is a schematic illustration of the PBF method using a metal powder with heterogeneous nucleation site particles. By inoculating the heterogeneous nucleation site particles with the metal powder used for AM, the heterogeneous nucleation site particles are introduced into the melt pool. Since solidification occurs in a large number of randomly orientated heterogeneous solidification nuclei particles at the heterogeneous nucleation site as scaffolds, it is expected that the grains will be refined and the texture will be eliminated [9,11,13]. Furthermore, because the solidification phenomenon does not occur locally but in a dispersed manner, it is expected that the structure will be homogenized, leading to improved formability [9,11,13].

The most common materials manufactured through metal AM are SUS316L austenitic stainless steel (Fe-17 mass% Cr-11 mass% Ni-2 mass% Mo) [11,14,15,16]; Ti-6Al-4V [9,17,18,19]; Inconel 718 (Fe-50~55 mass%Ni-17~21 mass%Cr-4.75~5.5 mass%Nb-2.8~3.3 mass%Mo) [20,21]; Al-10mass% Si-Mg alloys [22,23,24]; and Co-Cr-Mo alloys [25,26]. Out of these materials, SUS316L is selected as the obvious choice because it is manufactured through nearly all available AM routes. The reasons for SUS316L’s adaptability to different AM routes are its easy availability of feedstock in different forms, such as wire, powder, and sheets; excellent weldability due to low-carbon content; and efficient absorptivity with different melting sources, such as laser, electron beam, and electric/plasma arc [16]. Gonzalez et al. [27] have studied the influence of aging at 650 °C for 1000 h on the formation of embrittling phases, such as sigma, in SUS316L manufactured using wire arc DED. The sample was found to form secondary phases at the grain boundaries, but these phases caused negligible changes in the tensile properties of SUS316L. The microstructure and mechanical property correlation of SUS316L manufactured through laser PBF, electron beam PBF, and wire arc DED methods was assessed by Kumar et al. [16]. However, most of the studies on DED were of the wire arc type, and there are not many reports on the powder type.

In our study, the effect of SrO heterogeneous nucleation site particles on the formability, microstructure, and mechanical properties of AMed SUS316L stainless steel samples manufacture by the laser-based PBF method has been investigated [11]. The amount of SrO heterogeneous nucleation site particle was fixed to be 0.3 vol%. The relative densities of the samples produced with SrO under medium and low energy density conditions were found to be higher than those manufactured without SrO, although the addition of SrO negatively affected the relative density under the high energy density condition. The addition of SrO was shown to result in significant grain refinement on the SUS316L stainless steel alloy during laser-based PBF. It can be concluded that the addition of SrO heterogeneous nucleation site particles is a potential way to control the microstructure and mechanical properties of SUS316L samples manufactured by laser-based PBF [11]. Tan et al. [28] have studied the inoculation effects of TiN nanoparticles on the microstructure and mechanical properties of SUS316 manufactured by laser-based PBF. It is found that addition of 1 mass% TiN nanoparticles led to complete columnar-to-equiaxed transition and significant refinement of the austenite grains to ∼2 μm in the SUS316. However, most of the studies on inoculated SUS316L were carried out by laser-based PBF, and there are not many reports on DED. Meanwhile, the productivity of laser-based DED has been reported to be five times higher than that of laser-based PBF [29]. The laser diameter in laser-based DED is typically an order of magnitude larger than that in laser-based PBF, resulting in a larger melt pool, which is expected to result in a lower cooling rate [29]. Therefore, laser-based DED is expected to result in weak formation of a columnar structure. However, it is important to manufacture the SUS316L sample with a finer, uniform, and equiaxed microstructure using the DED method. Two different types of powder DED machine were chosen: a single-beam type machine specialized in high-speed manufacturing and multi-beam type machine specialized in precision manufacturing. The residence time in the liquid state of the melt pool formed by each machine is expected to be different, which may influence the heterogeneous nucleation performance of the SrO particles. In this study, the effects of the inoculation of the SrO heterogeneous nucleation site particles on the microstructure of AMed SUS316L are studied by two different DED machines with different melting states, including temperature, cooling conditions, and re-heating. In addition, the microstructure of the samples manufactured by DED was compared with that produced with other AM methods, especially those manufactured by the application of powder metallurgy.

## 2. Lattice Matching Between Heterogeneous Nucleation and the Crystallized Phase

Classical nucleation theory describes nucleation as a process driven by the competition between the volume free energy that favors growth and the interfacial free energy that opposes it. The heterogeneous nucleation site decreases the driving force required for nucleation. When the lattice matching between the crystalized phase and the heterogeneous nucleation site phase is better, the interfacial energy between them is reduced. The lattice matching between the heterogeneous nucleation site and the crystallized phase is evaluated using the parameter *M* [30,31]. The parameter *M* is approximately proportional to the specific misfit strain energy, and is defined as

*M* = *ε*_x_^2^ + *ε*_y_^2^ + (2/3)*ε*_x_*ε*_y_, (1)

where *ε*_x_ and *ε*_y_ are the principal misfit strains calculated using

*ε*_x_ = (|*x_hetero_*|*a_hetero_* − |*x_cryst_*|*a_cryst_*)/(|*x_cryst_*|*a_cryst_*) (2)

*ε*_y_ = (|*y_hetero_*|*a_hetero_* − |*y_cryst_*|*a_cryst_*)/(|*y_cryst_*|*a_cryst_*) (3)

where *x_hetero_* and *y_hetero_* are the principal directions of the heterogeneous nucleation site, and *x_cryst_* and *y_cryst_* are the principal directions of the crystallized phase. When the disregistry [32] and the planar disregistry [33] are calculated, which are often used in the solidification of metals, it is common to use the solidified phase as the denominator. Therefore, in this study, the denominator is chosen to be the solidified phase. It has been shown that the epitaxial relationship with a smaller *M* value becomes the preferred relationship. The parameter *M* is also used to predict the effective heterogeneous nucleation site materials, where the favorable heterogeneous nucleation site phase should have a small *M* value between crystallized phases [34,35]. If the *M* value is less than about 38 × 10^−3^, the nucleating agent is potent [35].

**Figure 3:**
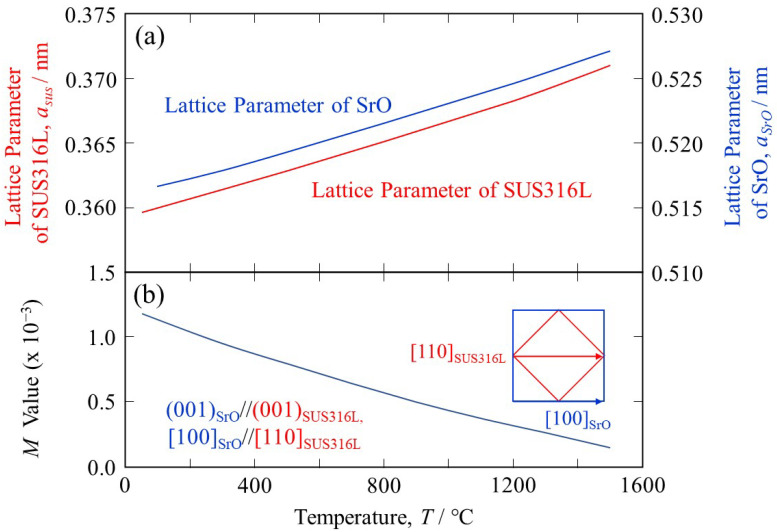
The temperature dependence of (**a**) the lattice parameters of SUS316L and SrO and (**b**) the *M* value between the SrO heterogeneous nucleation site and SUS316L under (001)_SrO_//(001)_SUS316L_ and [100]_SrO_//[110]_SUS316L_.

SUS316L has been reported to have a lattice parameter of approximately 0.35963 nm at room temperature [36]. The temperature dependence of the thermal expansion coefficient of SUS316L during the PBF-LB process is relatively small between room temperature and 1000 °C [37]. The thermal expansion coefficient of SUS316L at 10 °C is 17.62 × 10^−6^ °C^−1^, at 100 °C is 17.87 × 10^−6^ °C^−1^, at 300 °C is 18.2 × 10^−6^ °C^−1^, at 500 °C is 19.34 × 10^−6^ °C^−1^, at 800 °C is 20.86 × 10^−6^ °C^−1^, at 1200 °C is 21.61 × 10^−6^ °C^−1^, and at 1500 °C is 25.3 × 10 ^−6^ °C^−1^ [38]. On the other hand, the lattice parameter of SrO is 0.51615 nm [39], where the temperature dependence of the thermal expansion coefficient of SrO is reported by Reeber and Wang, and 11.67 × 10^−6^ °C^−1^ at 27 °C, 12.36 × 10^−6^ °C^−1^ at 127 °C, 12.80 × 10^−6^ °C^−1^ at 227 °C, and 16.82 × 10^−6^ °C^−1^ at 1727 °C, for example [40]. Using these data, the temperature dependence of the lattice parameters of SUS316L and SrO can be calculated, and the results are shown in Figure 3a.

The *M* values at the elevated temperatures can be calculated using the lattice parameters of SrO and SUS316L under the Baker–Nutting orientation relationship [41], i.e., (001)_SrO_//(001)_SUS316L_ and [100]_SrO_//[110]_SUS316L_, and the results are shown in Figure 3b. It is seen that the lattice matching is enhanced at elevated temperatures because the principal misfit strains calculated by Equations (2) and (3) have positive values (the atomic space of SrO is larger than that of SUS316L). The *M* value between SrO and SUS316L at 1500 °C is 0.1 × 10^−3^, which is much lower than 38 × 10^−3^. Therefore, we can conclude that SrO acts as a good heterogeneous nucleation site for solidification of SUS316L.

## 3. Experimental Procedure

### 3.1. Materials

In this study, two types of SUS316L powders were used. One of them is larger powder with a particle size of 45 μm to 150 μm (Sanyo Special Steel Co., Ltd., PSS316L, Himeji, Japan), and the other is smaller powder with a particle size of 25 μm to 53 μm (Daido Steel Co., Ltd., DAP-AM SUS316L, Nagoya, Japan), both prepared by gas atomization. The chemical compositions of the SUS316L powders used are given in Table 1. The chemical composition of the larger SUS316L powder was determined using the combustion infrared absorption method for C and S, while X-ray fluorescence was determined for Ni, Cr, Mo, Mn, Si, and P. However, that of smaller powder was determined by X-ray fluorescence. As heterogeneous nucleation site particles, SrO particles of a size of 2 μm (*D_10_*) to 20 μm (*D_90_*) [11] (Kojundo Chemical Laboratory Co., Ltd., SrO Powder, Sakado, Japan) were used. X-ray diffraction (XRD) analysis with copper Kα radiation was performed on the SUS316L and SrO powders using an X-ray diffractometer (Shimadzu, LabX XRD-6100, Kyoto, Japan) to identify the phases in the SUS316L sample and determine the lattice constants of the SUS316L and SrO used. The measurement range was 2*θ* = 25–100°.

Powder mixing was performed with a 3D motion mixer (WAB-GROUP, 3D SHAKER MIXER TURBULA T2F, Basel, Switzerland) [42,43] for 1 h. The motion of the container during mixing consisted of two rotations of the container around its longitudinal axis and horizontal translation to achieve uniform mixing of the particles. The volume fraction of the SrO heterogeneous nucleation site particles in the mixed powder of the SUS316L/SrO particles has been set to 0.3 vol% for the DED studies, since the addition level of the SrO particles was 0.3 vol% for the PBF study [11]. On the other hand, the levels of addition of SrO particles for flowability studies were 0.1 vol%, 0.3 vol %, and 1.0 vol%.

Flowability is one of the most important properties of the powder for DED, since the manufacturing process begins with the flow of powder streaming and laser–material interactions, which further influence the molten pool dynamics and formation of defects [44]. The flowability of the powder is dependent on a set of parameters, one of which is the repose angle. The powder will flow at angles greater than the repose angle. The repose angles of the SUS316L powder with and without SrO particles were measured using the fixed-funnel method [45].

### 3.2. DED Manufacturing

In this study, two types of DED machines were used to manufacture the SUS316L samples with and without the SrO heterogeneous nucleation site particles. One is the LAMDA 200 machine made by Nidec Machine Tool Corp. (Ritto, Japan), formerly Mitsubishi Heavy Industries Machine Tool Co., Ltd. The other is a multi-beam DED-LB system (Muratani Machine Inc., ALPION Series, Fine-Laser Cladding System, Kanazawa, Japan).

#### 3.2.1. LAMDA 200 Machine

**Figure 4:**
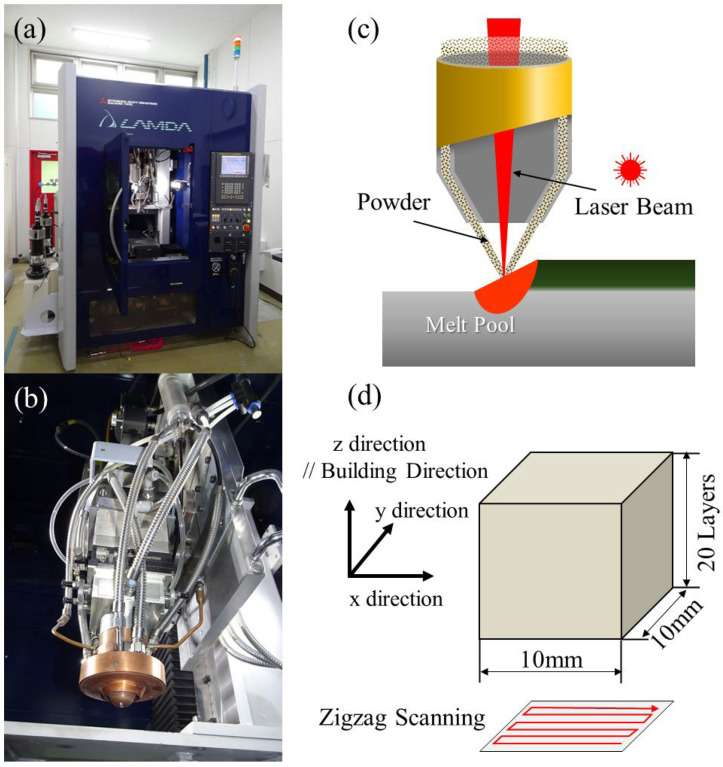
(**a**) LAMDA 200 machine. (**b**) A photograph and (**c**) schematic illustration of the head. (**d**) Size of the block-shaped sample and scanning strategy.

The LAMDA 200 machine, shown in Figure 4, employs a 2 kW fiber laser with a 1070 nm wave length and offers a maximum manufacturing size of 200 mm × 200 mm × 200 mm [24,46,47,48]. A full photograph of the LAMDA 200 machine is shown in Figure 4a. The manufacturing speed is about 10 times faster than that of the PBF method. Figure 4b and Figure 4c show a photograph and schematic illustration of the head in the LAMDA 200 machine, respectively. The head in the LAMDA 200 machine is equipped with a powder feeding nozzle on its tip, which accurately feeds metal powder to the laser focusing position on the substrate. The nozzle used in this study is a 3-port feeding type. A local shield has been installed to prevent the oxidation of active metals during manufacturing.

Block-shaped SUS316L samples with a width and length of 10 mm and comprising 20 layers, shown in Figure 4d, were manufactured. The processing parameters are a laser power of *P* = 400 W, 600 W, 800 W, or 1000 W; a laser scanning speed of *v* = 13.3 mm/s; a hatch spacing (feed pitch) of *h* = 1 mm; and a layer thickness (z pitch) of *t* = 0.5 mm. The volumetric energy densities, defined by *P*/*vht*, are 60, 90, 120, and 150 J/mm^3^, respectively. The scanning strategy was zigzag scanning, by which the scanning direction was changed at the corner of the sample, as shown in Figure 4d. The process parameters, except for the layer pitch for the LAMDA experiments, are the same as in the previous study [48], by which a 20 mm × 10 mm × 10 mm SUS316L sample was manufactured.

#### 3.2.2. ALPION Series Machine

**Figure 5:**
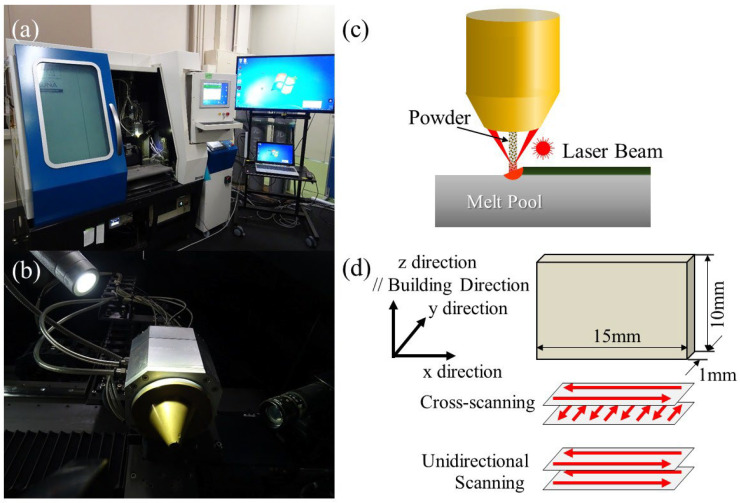
(**a**) ALPION Series machine. (**b**) A photograph and (**c**) schematic illustration of the head. (**d**) Size of the block-shaped sample and scanning strategies.

The other DED experiments were performed by the ALPION Series machine, shown in Figure 5. Figure 5a shows an overall photograph of the ALPION Series machine. Figure 5b and Figure 5c show a photograph and schematic illustration of the head in the ALPION Series machine, respectively. The system is equipped with six semiconductor lasers with a wavelength of 975 nm and a maximum power output of 50 W (the maximum power output of 300 W in total six lasers) [49,50,51]. The ALPION Series machine has been developed to realize precise and minimal heat-affected zones.

To investigate the effect of adding heterogeneous nucleation site particles to the microstructure of SUS316L, SUS316L samples were manufactured with a plate shape of 15 mm in length, 1 mm width, and 10 mm height, as shown in Figure 5d. Here, manufacturing was performed with a total laser power of 150 W, a scanning speed of 10 mm/s, a hatch spacing of 0.2 mm, a layer thickness of 0.15 mm, and the number of layers was 68. The volumetric energy density is 500 J/mm^3^. Two different scanning strategies were used, as also shown in Figure 5d. One is cross-scanning, by which the scanning direction of odd layers is the x direction, while the y direction in the even layers. Another method is unidirectional scanning, by which the scanning direction is fixed to the x direction for all layers.

In the ALPION Series machine experiment, the process parameters were selected to minimize the formation of voids within the samples [49]. It is known that the larger laser power in the ALPION Series machine experiment results in a wider bead width, and the larger powder feed rate results in a higher bead height. Based on these facts, in this study, the laser power and the powder feed rate were selected to minimize the formation of voids due to gaps between the beads during manufacturing and voids within the individual beads. Single-track preliminary tests were conducted using uninoculated SUS316L powder on a SUS316L base plate to explore stable conditions for good adhesion and minimal defects. The process parameters ranged from 90 to 210 W (every 30 W) in laser power and 5, 10, 15, and 20 mm/s in scanning speeds. As a result, 150 W and 10 mm/s were determined to be in the center of the stable region and adopted as manufacturing conditions. To enhance internal gas release within each bead, a slower powder feed rate was selected. However, since feed rates below 10 mg/s became unstable during powder feeding, the feed rate of 10 mg/s, the lower limit for stable feed, was selected. Furthermore, based on the single bead height obtained under these conditions, the layer thickness was set at 0.15 mm, and the hatch spacing was set at 0.2 mm, which is half of the bead width, so that each bead overlapped by 50%. The process parameters for both machines are summarized in Table 2.

### 3.3. Characterization of Manufactured Samples

The cross sections of the manufactured samples were wet-polished using emery paper and then buffed with diamond paste and colloidal silica slurry to a mirror finish. The microstructural observation of the samples was carried out using a scanning electron microscope (SEM; JEOL, JSM-7001F, Akishima, Japan) and electron backscattered diffraction (EBSD; AMETEK Inc., ORION, Pleasaton, CA, USA). To remove the low-quality orientation data obtained by EBSD, the measurement data with confidence index (CI) value of more than 0.1 was used. Furthermore, O, Fe, Sr, Ni, and Cr images of the particle observed in the inoculated SUS316L sample manufactured by the ALPION Series machine were performed using an SEM (JEOL, JSM-7001F) with an energy dispersive X-ray spectroscopy (EDS; Thermo-Fishier Science K.K., Ultradry, Waltham, MA, USA) detector. For the particulate structure showing a high Sr content, detected by EDS analysis, a cross-sectional thin-film specimen was prepared using a focused ion beam (FIB) system and examined by transmission electron microscopy (TEM). The FIB system used was JEOL, JIB-4700F. TEM observations were performed using JEOL JEM-2100Plus at 200 kV. For the electron diffraction patterns obtained by TEM, all camera constants were set to the same values, and the images were saved at the same size (number of pixels). ImageJ software (1.54g) was used to measure the distances between the diffraction spots within each image.

In this study, defects in the inoculated and uninoculated SUS316L samples manufactured by the LAMDA 200 machine are evaluated in 3D using X-ray computed tomography (X-ray CT). The use of X-ray CT allows the analysis of the size and spatial distribution of pores without damaging the samples. X-ray CT was applied using a microfocus X-ray device (Shimadzu, InspeXio SMX-225CT, Kyoto, Japan) to visualize internal defects within the manufactured samples. The sample was placed on a rotating stage, and original 2D X-ray images were acquired at various angles around the rotated object. In this study, scans with voxel sizes of 38 μm before trimming and 33 μm after trimming were performed. From the obtained sectioned data, 3D images indicating the internal defects were reconstructed using VGSTUDIO MAX (Volume Graphics, Heidelberg, Germany).

**Figure 6:**
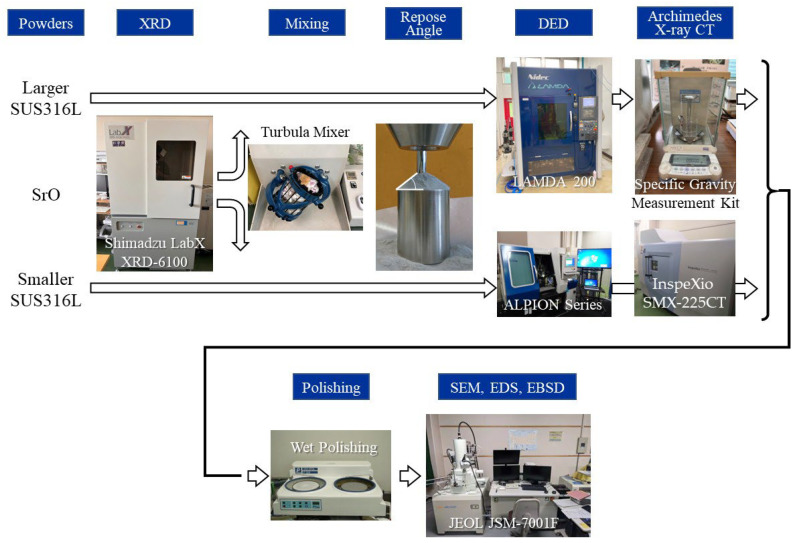
Flow chart of experimental procedures.

The density of the inoculated and uninoculated SUS316L samples manufactured by LAMDA 200 machine was measured using the Archimedes method (Shimadzu, SMK-301, Kyoto, Japan). The sample was weighed in air and on a suspender in distilled water and then the density was evaluated using the density of water at that temperature. This measured density value was compared to the theoretical value of SUS316L and the relative density was calculated. The abovementioned experimental method is summarized in Figure 6.

## 4. Results

### 4.1. Powders

**Figure 7:**
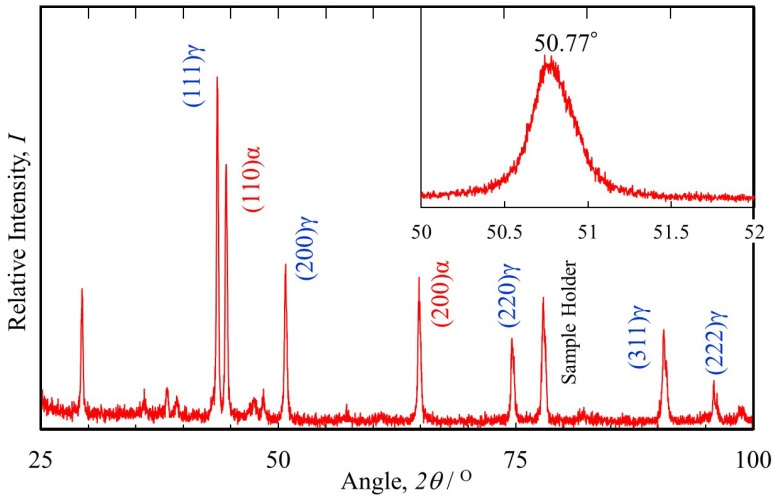
The result of the XRD experiment for the SUS316L powder. Detailed investigation of the 2*θ* range between 50° and 52° is also shown to the upper right of the figure.

The XRD experiments were conducted to investigate the presence of phases in SUS316L powder and lattice constants of SUS316L and SrO. The result of SUS316L is shown in Figure 7. (111)_γ_, (200)_γ_, (220)_γ_, (311)_γ_, and (222)_γ_ peaks of the fcc phase as well as (110)_α_ and (200)_α_ peaks of the bcc phase could be observed. It is found that the powder used shows not only the austenite phase with fcc structure but also ferrite or martensite phases with bcc structure. The results of a detailed investigation of the 2*θ* range between 50° and 52° are shown to the upper right of the figure. Using the Rietveld method, the lattice constant of SUS316L was determined to be a = 0.3593 nm from the (200)_γ_ peak at 2*θ* = 50.77°. The lattice constant of SrO was similarly investigated using the Rietveld method and found to be 0.5158 nm. Since the Baker–Nutting orientation relationship is expected between SrO and SUS316L, the [100]_SrO_ direction is parallel to the [110]_SUS_ direction. The lattice constant of SUS316L, 0.3593 nm, multiplied by the square root of 2 becomes 0.5081 nm, which is close to the lattice constant of SrO, 0.5158 nm. Therefore, SrO effectively acts as a heterogeneous nucleation site for SUS316L.

**Figure 8:**
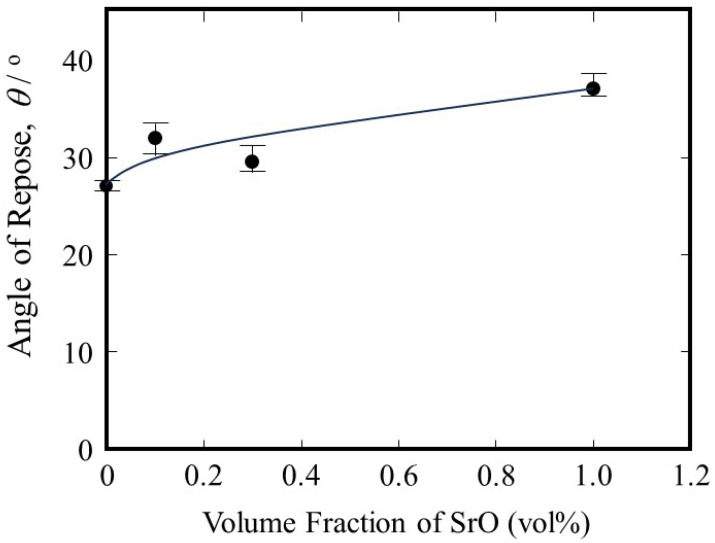
The angle of repose of the SUS316L powder with SrO heterogeneous nucleation site particles.

Figure 8 is the repose angle of the SUS316L powder with SrO heterogeneous nucleation site particles plotted against the volume fraction of SrO particles. It is seen that the repose angle increases with an increase in the volume fraction of the SrO particles. According to the Carr classification of the flowability of powders based on the repose angle [52,53], a repose angle of less than 30° is classified as very free-flowing, repose angles of 30° to 38° are classified as free-flowing, and repose angles of 38° to 45° are classified as having fair to passable flow. Therefore, the flowability of the SUS316L powders with 0.0 vol% 0.1 vol% and 0.3 vol% are classified as very free-flowing, free-flowing and free-flowing, respectively, with fair to passable flow for 1.0 vol% SrO particles. The volume fraction of SrO particles should be smaller than 1.0 vol%. In addition, the addition of a small amount of SrO particles may emphasize the negative effect of SrO particle addition. In this study, therefore, the volume fraction of SrO heterogeneous nucleation site particles in SUS316L powder is fixed to be 0.3 vol%.

### 4.2. SUS316L Samples Manufactured by the ALPION Series Machine

**Figure 9:**
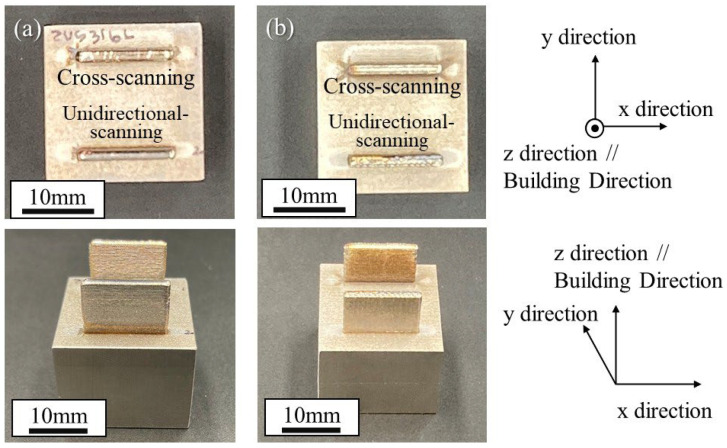
The appearance of (**a**) uninoculated and (**b**) inoculated samples manufactured by the ALPION Series machine. The upper and lower samples shown in each photograph were manufactured by cross-scanning and unidirectional scanning, respectively.

Figure 9a and Figure 9b show the appearance of the uninoculated and inoculated samples manufactured by the ALPION Series machine, respectively. The upper and lower samples shown in each photograph were manufactured by cross-scanning and unidirectional-scanning methods, respectively. As can be seen, wall samples were successfully manufactured by the ALPION Series machine using SUS316L samples with and without SrO heterogeneous nucleation site particles under different scanning strategies. Using these samples, microstructural observations were carried out on both the x–z plane and the y–z plane.

**Figure 10:**
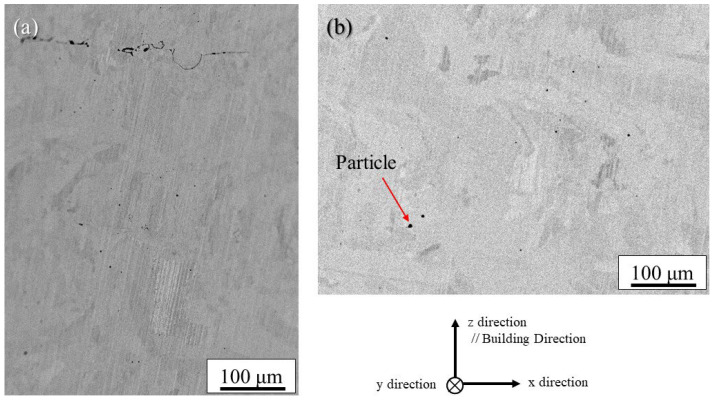
BSE images of the (**a**) uninoculated and (**b**) inoculated SUS316L samples manufactured under the cross-scanning strategy observed in the x–z plane.

Figure 10a and Figure 10b show the backscattered electron (BSE) images of the uninoculated and inoculated SUS316L samples manufactured under the cross-scanning strategy observed in the x–z plane, respectively. It should be noted that the uninoculated SUS316L sample has plate-shaped defects that extend horizontally. These defects are thought to be areas that have not melted during lamination. Although such defects were also observed in the SrO-inoculated samples, the number of defects was smaller than that of the uninoculated samples. Instead, the inoculated SUS316L sample contains some small particles, indicated by an arrow, as shown in Figure 10b.

**Figure 11:**
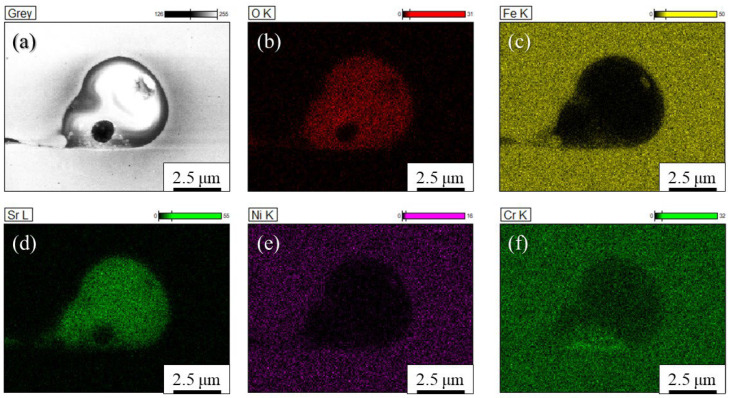
(**a**) SEM view and (**b**) O, (**c**) Fe, (**d**) Sr, (**e**) Ni, and (**f**) Cr images of the particle observed in the inoculated SUS316L sample manufactured by the ALPION Series machine.

Detailed observations of such particles observed in the inoculated SUS316L sample manufactured using the cross-scanning strategy (Figure 10b) were carried out. Figure 11a, Figure 11b, Figure 11c, Figure 11d, Figure 11e and Figure 11f show an enlarged SEM view of the particle, O, Fe, Sr, Ni, and Cr images of the particle by SEM with EDS, respectively. As the figures show, it is clear that the particle shows a large amount of Sr and O, but there is also a somewhat large amount of Cr. While the results are not presented here, diffraction peaks corresponding to the γ phase and the α phase were observed in the SUS316L sample manufactured by the ALPION Series machine, while diffraction peaks attributable to SrO were not detected. This is considered to be due to the very low volume fraction of SrO. Furthermore, since no additional crystalline peaks were identified, the presence of any Sr-containing phases other than SrO in significant amounts can be excluded. Based on these observations, it is more reasonable to consider that the particles analyzed in Figure 11 are the original SrO powder that was mixed in, rather than a new phase that was transformed during manufacturing. Therefore, the particles found in Figure 10b can be identified as the SrO heterogeneous nucleation site particles. Also, because the morphology is not particulate, it is possible that the particle melted during the process.

**Figure 12:**
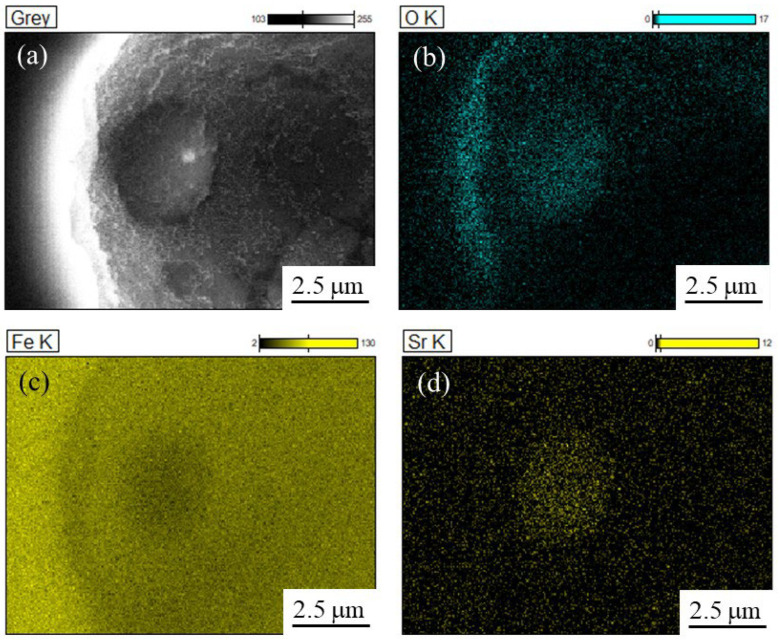
(**a**) SEM view and (**b**) O, (**c**) Fe, and (**d**) Sr images of the particle observed in the inoculated SUS316L sample manufactured by the ALPION Series machine.

Figure 12a, Figure 12b, Figure 12c and Figure 12d also show the SEM image, O, Fe, and Sr images, respectively, obtained using the JSM-7001F and EDS Ultra Dry. Similarly to Figure 11, the particle with a 3 μm grain is captured in the SEM image and contains high amounts of Sr and O.

**Figure 13:**
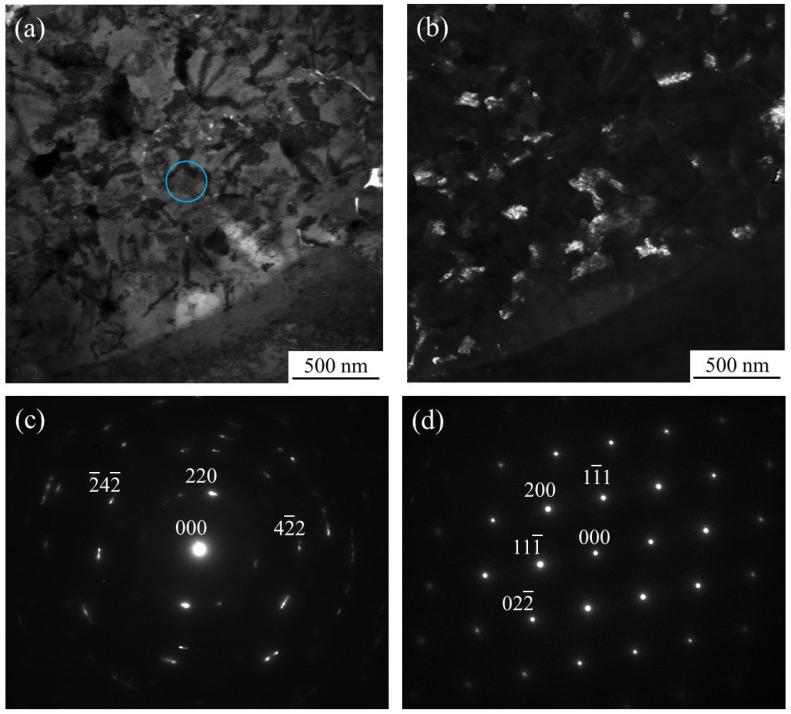
(**a**) Bright-field image, (**b**) dark-field image, (**c**) electron diffraction patterns obtained from light-blue circle region (particle region) in (**a**), and (**d**) electron diffraction patterns obtained from lower-right region (matrix region) in (**a**). The sample is the inoculated SUS316L sample manufactured by the ALPION Series machine.

For the particle showing a high Sr content, detected by EDS analysis, TEM observations were performed. Figure 13a and Figure 13b show a bright-field image and a dark-field image, respectively. The electron diffraction patterns obtained from the light-blue circle region (particle region) and the lower-right region (matrix region) in Figure 13a are shown in Figure 13c and Figure 13d, respectively. The dark-field image in Figure 13b was obtained using the [−2 4 −2] spot from the electron diffraction pattern in Figure 13c. Note that Figure 13d was obtained by tilting the sample after photographing pattern shown in Figure 13c.

It can be seen from Figure 13b that it is composed of fine particles with particle sizes of approximately 50 to 200 nm. Figure 13c and Figure 13d were analyzed and indexed as fcc (z = [−1 1 3]) and fcc (z = [0 1 1]), respectively. Furthermore, the lower-right region of Figure 13a exhibited the same crystal orientation as the electron diffraction pattern, similar to that of Figure 13d. Therefore, Figure 13d is considered to represent austenite. Since Figure 13c,d are the electron diffraction patterns obtained with the same camera length, the distances from 000 to 022 in each image are inversely proportional to the lattice constants of the phases from which the respective diffraction patterns were acquired. The distances from spot 000 to spot 002 in Figure 13c and Figure 13d are denoted *l*_(*c*)_ and *l*_(*d*)_, respectively, and can be estimated as *l*_(*c*)_/*l*_(*d*)_ ≈ 0.634. The lattice parameter ratio of *a_SUS_*/*a_SrO_* is 0.697, which is a sufficiently close approximation to the values possible for the phases contained within this structure. On the basis of the two abovementioned points (the ratio of spot distances in the electron diffraction pattern and the fact that the target particles exhibit an fcc atomic arrangement), the particles observed in this study can be identified as SrO.

**Figure 14:**
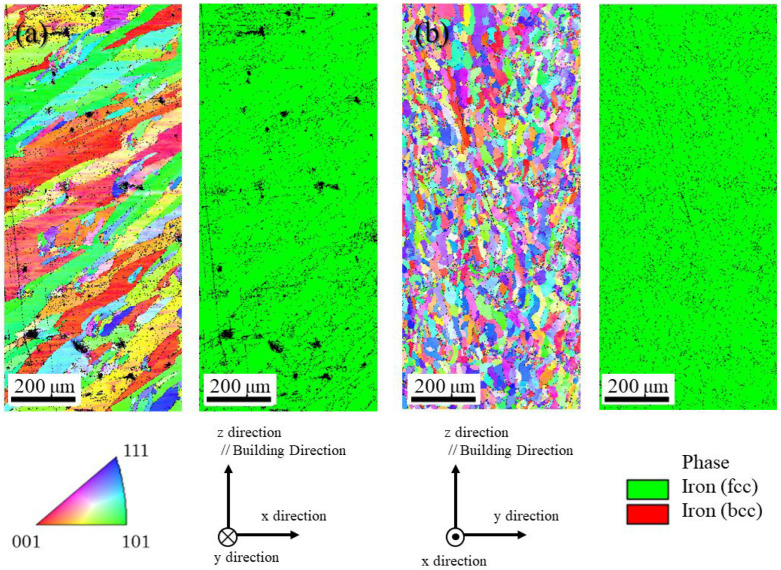
The microstructure of the uninoculated SUS316L sample manufactured under the unidirectional scanning method observed on (**a**) x–z plane and (**b**) y–z plane. In each figure, the left and right figures are the IPF map and the phase map, respectively.

The microstructure of the uninoculated SUS316L sample manufactured under the unidirectional scanning strategy observed on the x–z plane and y–z plane is shown in Figure 14a and Figure 14b, respectively. In each figure, the left and right figures are the inverse pole figure (IPF) map and the phase map, respectively. From the phase maps, only the austenite phase can be observed, although the powder used shows not only the austenite phase with the fcc structure but also the ferrite or martensite phases with the bcc structure, as shown in Figure 7. This is because the DED process involves the melting and solidification of the metal, unlike the processes by application of powder metallurgy, as shown in Figure 1c. The difference in the microstructure of the SUS316L samples manufactured by the application of powder metallurgy will be discussed later.

Since the sample thickness in the y direction is thin, the microstructure observed in the y–z plane shown in Figure 14b is relatively fine. It is interesting to note here that the microstructure observed on the x–z plane shows course and columellar grains, as shown in Figure 14a. In this way, the sample manufactured by the unidirectional scanning strategy has an elongated microstructure oriented at an angle in a specific direction. Furthermore, fine grains less than 10 μm cannot be observed, while many voids were observed, which can be seen as black dots in the SEM image. This microstructure might be improved by the addition of SrO heterogeneous nucleation site particles.

**Figure 15:**
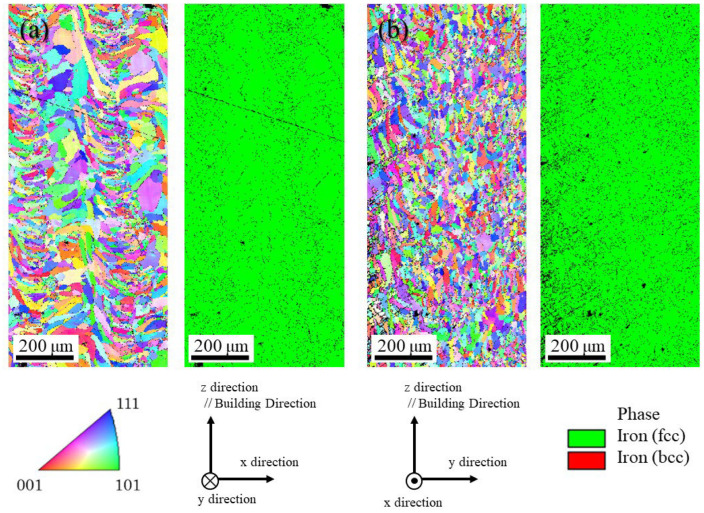
The microstructure of the uninoculated SUS316L sample manufactured under the cross-scanning strategy observed in (**a**) the x–z plane and (**b**) the y–z plane. In each figure, the left and right figures are the IPF map and the phase map, respectively.

Figure 15a and Figure 15b show the microstructures of the uninoculated SUS316L sample manufactured under a cross-scanning strategy observed on the x–z plane and the y–z plane, respectively. In each figure, the left and right figures are the IPF map and the phase map, respectively. From the phase maps, only the austenite phase can be observed, which is consistent with the results of the uninoculated SUS316L sample manufactured under the unidirectional strategy shown in Figure 14. Unlike the sample manufactured by the unidirectional method, a wavy microstructure can be seen parallel to the x direction of the scanning in the figure observed on the x–z plane, as shown in Figure 15a. Although the SrO heterogeneous nucleation site particles were not inoculated, a fine grain structure can be observed on the y–z plane, as shown in Figure 15b, but in some areas, columnar structures elongated in the building direction can also be seen. The left side of the photograph shows the microstructure near the center part of the plate-shaped sample, namely far from the surface. In this area, the formation of many columnar crystals can be observed. In addition, some of these reflect the shape of the molten pool. Such a microstructure might be formed because it is easy for the heat to remain. However, cross-scanning may be an effective strategy for refining the structure, although the microstructure is not uniform, with some areas being fine and others being sparse.

**Figure 16:**
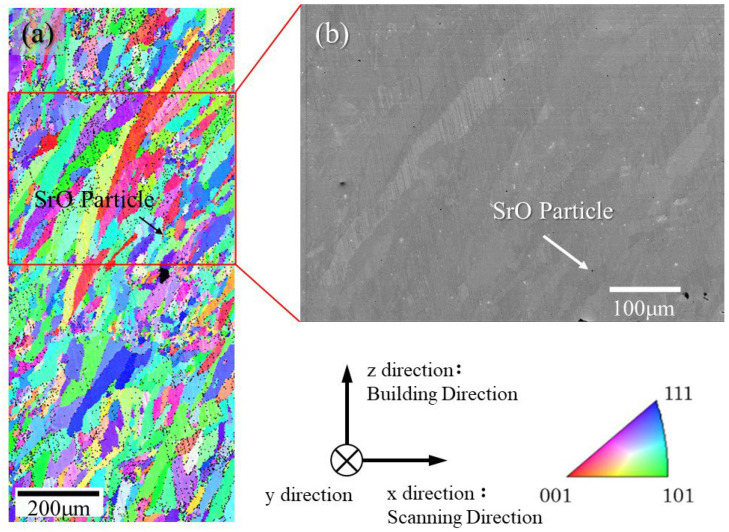
(**a**) IPF map and (**b**) BSE image of the SUS316L sample manufactured with 0.3 vol% SrO heterogeneous nucleation site particles under unidirectional scanning strategy. The observation plane is the x–z plane.

Figure 16a and Figure 16b show the IPF map and the BSE image of the SUS316L sample manufactured with 0.3 vol% SrO heterogeneous nucleation site particles under the unidirectional scanning strategy, respectively. The observation was carried out on the x–z plane. Composition analysis confirmed the presence of a SrO particle at the tip of the arrow. Most of the other black points in the SEM image are thought to be holes caused by defects. From the IPF map, the fine grain can be observed around the SrO heterogeneous nucleation site particle. Thus, the heterogeneous nucleation ability of SrO particles can be confirmed. The addition of SrO heterogeneous nucleation site particles can achieve a fine grain structure in the AMed SUS316L sample using the ALPION Series machine.

Using EBSD measurement data, the average grain sizes of the inoculated and uninoculated samples manufactured using the unidirectional scanning strategy were calculated, taking into account weighting by occupied area. Here, the observed plane was the x–z plane. The lengths of the long and short axes of each grain were measured and averaged. For curved grains, the longest side along the curve was defined as the “long side”, and the length perpendicular to the long side was defined as the “short side”. The aspect ratio determined by the long axis of the grain divided by the short axis is also calculated. The results are listed in Table 3. The standard deviation (*SD*) and the number of grains analyzed (*N*) are also shown in this table. As can be seen, the crystal grains of the SUS316L sample with the addition of SrO heterogeneous nucleation site particles are refined compared to the sample without SrO particles. In addition, the aspect ratio of the inoculated sample, especially fine grains, is smaller than that of the uninoculated sample. In this way, the addition of particles tends to result in equiaxed crystals. It is concluded that the SrO heterogeneous nucleation site particles contribute to the refinement and equiax of the grain structure in the powder-type DED method.

### 4.3. SUS316L Samples Manufactured by the LAMDA 200 Machine

**Figure 17:**
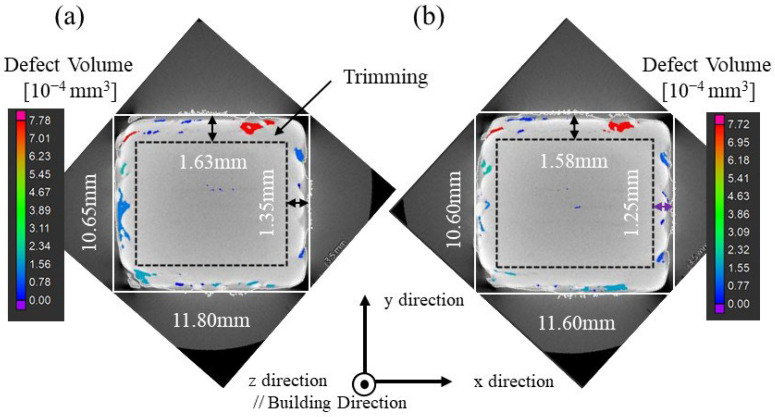
X-ray images of (**a**) uninoculated and (**b**) inoculated SUS316L samples manufactured at a laser power of 400 W.

Block-shaped SUS316L samples that were 10 mm in length and width and approximately 10 mm in height were manufactured by the LAMDA 200 machine. The results of X-ray CT of the uninoculated and inoculated SUS316L samples manufactured at a laser power of 400 W are shown in Figure 17a and Figure 17b, respectively. As shown in the figure, the surface of the manufactured samples contained many defects. Therefore, approximately 1.5 mm was cut off (trimming) the surface prior to the following material evaluation.

**Figure 18:**
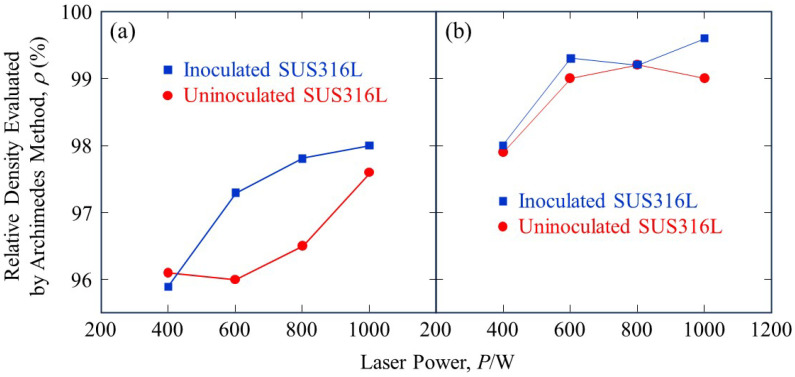
The relative density of (**a**) pre-trimmed and (**b**) trimmed SUS316L sample manufactured by the LAMDA 200 machine evaluated using the Archimedes method.

The relative densities of the SUS316L samples manufactured by the LAMDA 200 machine were evaluated using the Archimedes method and are shown in Figure 18. Figure 18a shows the relative density of the pre-trimming samples. Increasing the laser power shows an increase in the relative density. However, these values are strongly influenced by the defects located near the surface. Since it is impossible to evaluate the bulk state with this measurement, trimming of the samples was performed. Figure 18b shows the relative density after trimming. These figures show that trimming increases the relative density evaluated by the Archimedes method under all processing conditions, confirming that many defects were located near the surface.

**Figure 19:**
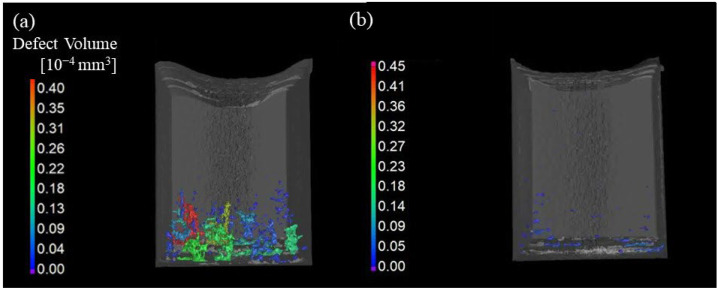
The results of X-ray CT of (**a**) uninoculated and (**b**) inoculated SUS316L samples manufactured by the LAMDA 200 machine.

Figure 19 shows the results of the X-ray CT of the samples manufactured with a laser power of 400 W and a scanning speed of 800 mm/min. The samples shown in Figure 19a and Figure 19b are uninoculated and inoculated SUS316L samples, respectively, after trimming. In the case of the uninoculated sample, plate-shaped defects parallel to the longitudinal direction of the scanning path were observed near the bottom surface. However, the formation of such defects was suppressed by the addition of SrO heterogeneous nucleation site particles. In addition, the size of the defects found in the inoculated sample is smaller than that in the uninoculated sample, which can be recognized by the color key. The defect volume ratio evaluated by X-ray CT was 0.46 vol% in the uninoculated sample and 0.04 vol% in the inoculated sample.

**Figure 20:**
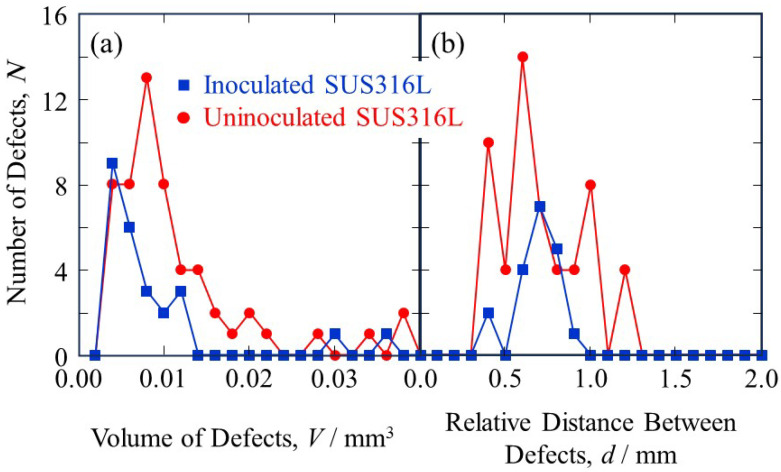
The number of defects in the LAMDA samples plotted against (**a**) the volume of defects and (**b**) the relative distance between the defects.

Figure 20a shows the number of defects in the LAMDA samples plotted against the volume of defects. The mean value, median, and standard deviation for the uninoculated SUS316L sample are 0.032 mm^3^, 0.009 mm^3^, and 0.062 mm^3^, respectively, while they are 0.009 mm^3^, 0.005 mm^3^, and 0.011 mm^3^ for the inoculated SUS316L sample, respectively. As shown in Figure 20a, the defect volume values in the uninoculated SUS316L sample are concentrated below 0.01 mm^3^. Furthermore, defects exceeding 0.015 mm^3^ decrease rapidly and the tail of the distribution extends to approximately 0.04 mm^3^. The presence of these large defects increases the mean value far above the median and increases the standard deviation. In the case of the inoculated SUS316L sample, the defect volume values are also concentrated below 0.01 mm^3^. However, the values are significantly decreased compared to the uninoculated SUS316L sample. In addition, defects exceeding 0.015 mm^3^ have almost completely disappeared, resulting in a very narrow distribution. This change in the distribution shape indicates that SrO is highly effective in suppressing the formation and growth of large defects and causing the remaining defects to be uniform in the fine state. Figure 20b shows the number of defects in the LAMDA samples plotted against the relative distance between the defects. The mean value, median, and standard deviation for the uninoculated SUS316L sample are 0.75 mm, 0.66 mm, and 0.32 mm, respectively, while they are 0.86 mm, 0.70 mm, and 0.42 mm for the inoculated SUS316L sample, respectively. Since the calculation of the relative distance between the defects uses a representative value of the position of the defect, it is not dependent on the size of the defect. Therefore, the relative distance between the defects corresponds to the layer thickness (0.5 mm) and the hatch spacing (1 mm) when the defects are formed based only on the scanning path. The distances for the uninoculated SUS316L sample are widely distributed, from approximately 0.4 mm to 1.2 mm, indicating that the defect positions are not uniform and the defects may have been formed due to various factors. For the inoculated SUS316L sample, the mean, median, and standard deviation are larger than those of the uninoculated SUS316L sample. This is thought to be due to the overall increase in the distance between defects due to the decrease in the number of defects. A wider distribution is found for the inoculated SUS316L sample, since the decrease in the number of defects has resulted in the presence of isolated defects that are far apart. In this way, the addition of SrO heterogeneous nucleation site particles improves formability and significantly suppresses the occurrence of defects.

**Figure 21:**
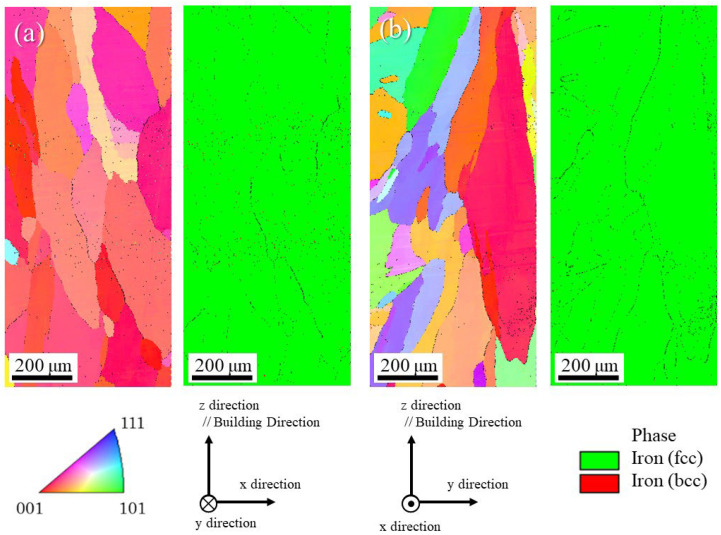
Microstructures of the uninoculated SUS316L sample manufactured using the LAMDA 200 machine: (**a**) observed in the x–z plane and (**b**) in the y–z plane. The images in the left figures show the IPF maps, and the images in the right figures show the phase maps.

**Figure 22:**
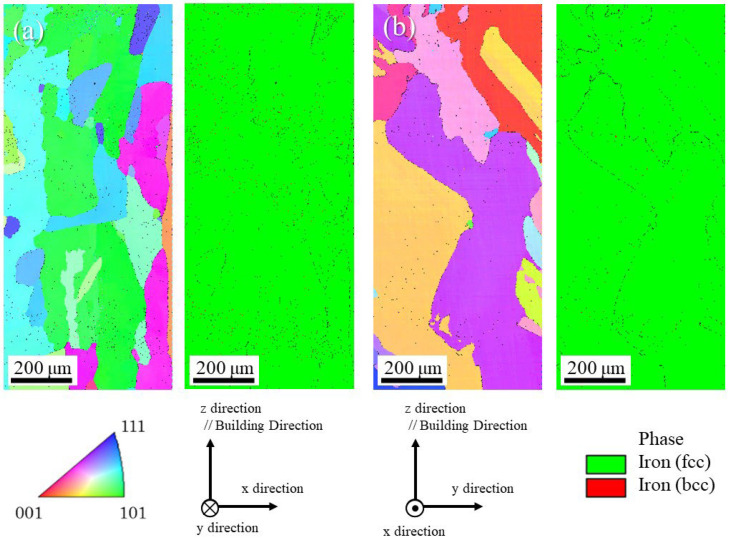
The microstructures of the inoculated SUS316L sample manufactured using the LAMDA 200 machine: (**a**) observed in the x–z plane and (**b**) in the y–z plane. The images in the left figures show the IPF maps, and the images in the right figures show the phase maps.

The microstructures of the uninoculated and inoculated SUS316L samples manufactured using the LAMDA 200 machine are shown in Figure 21 and Figure 22, respectively. In both figures, (a) is observed in the x–z plane and (b) in the y–z plane. The images in the left figures show the IPF maps, and the images in the right figures show the phase maps. Only the austenite phase can be observed from the phase maps, which is consistent with the results of the samples manufactured by the ALPION Series machine. In both samples, coarse grains are observed and no refinement as a result of the addition of SrO particles is evident. The average grain size and aspect ratio calculated using EBSD measurement data for the samples manufactured using the LAMDA 200 machine were listed in Table 4. This is thought to be because the samples were maintained at high temperatures by the repetition of laser irradiation, which causes the coarsening of the grains. However, when the crystal orientation was compared, the uninoculated sample showed a strong texture, whereas the crystals in the inoculated material are somewhat random.

**Figure 23:**
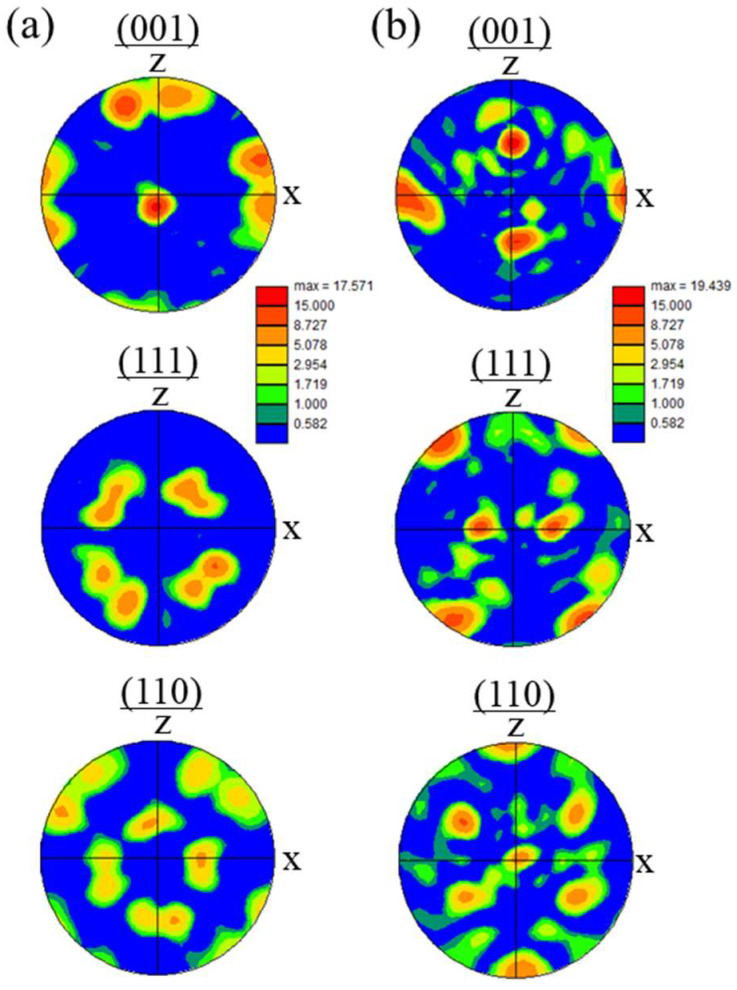
The (001), (111), and (110) pole figures of (**a**) the uninoculated SUS316L sample and (**b**) inoculated SUS316L sample.

To confirm the effects of the SrO particles on the crystallographic texture, (001), (111) and (110) pole figures of the uninoculated SUS316L and inoculated SUS316L samples are shown in Figure 23a and Figure 23b, respectively. From the pole figures in Figure 23a, it is seen that the uninoculated SUS316L sample has a strong crystallographic texture because only two kinds of crystal orientations are observed. However, although the inoculated SUS316L has some strong peaks due to the large grains, many peaks are observed, as shown in Figure 23b. Hence, compared to the uninoculated SUS316L sample and the inoculated SUS316L sample, the crystallographic texture of the inoculated SUS316L sample is weaker than that of the uninoculated SUS316L sample. Usually, the crystallographic texture of the solidified sample is formed by a temperature gradient. However, if there are heterogeneous nucleation particles in the melt and these particles act as the heterogeneous nucleation site, a strong crystallographic texture cannot be formed. This is because crystal nucleation starts with heterogeneous nucleation particles. In this way, the addition of heterogeneous nucleation site particles also works to suppress the formation of the texture.

## 5. Discussion

In the previous section, the effect of the addition of SrO heterogeneous nucleation site particles on the microstructures of samples manufactured by two different DED machines was studied. In the following sections, these microstructures will be compared with those manufactured by the process based on sintering (application of powder metallurgy).

### 5.1. SUS316L Samples Manufactured by Studio System^TM^ Technology

**Figure 24:**
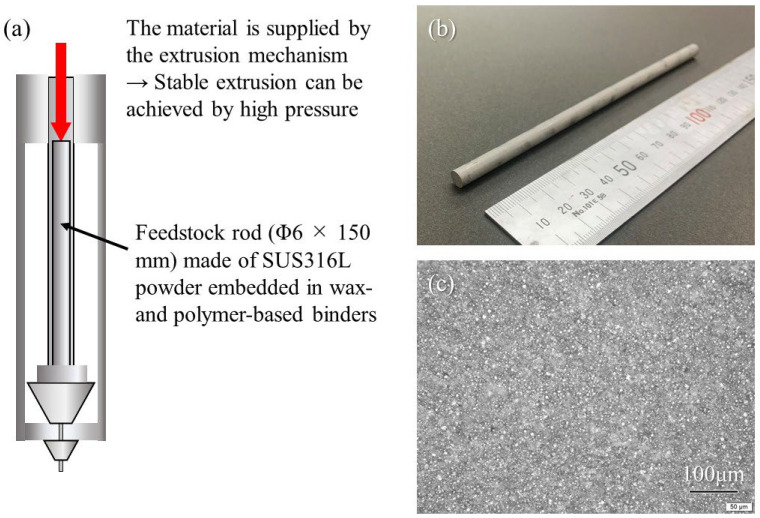
(**a**) Schematic illustration of Studio System^TM^ technology. (**b**) Macrostructure and (**c**) microstructure of the feedstock rod made of SUS316L powder embedded in wax- and polymer-based binders.

The SUS316L sample was manufactured using the material extrusion (MEX) method with a sinter-based extrusion process using Studio System^TM^ technology (Desktop Metal, DM, Burlington, MA, USA) [54], as shown in Figure 24. In this method, MIM shares the same debinding and sintering processes, except for the green part production method. The printer uses cartridges containing the feedstock in the form of Φ6 × 150 mm rods made of SUS316L powder embedded in wax- and polymer-based binders [14], as shown in Figure 24a,b. Because the material is supplied by the extrusion mechanism, stable extrusion can be achieved under high pressure. In addition, because there is no need to bend the rod during the process, the metal powder can be dispersed at a high concentration within the rod, as shown in Figure 24c. The bars are extruded through an extrusion head (containing a heated nozzle at a temperature of 160 to 180 °C) [54]. After additive manufacturing of the sample was performed, the removal of the binder (debinder) from the green part was carried out, followed by sintering.

**Figure 25:**
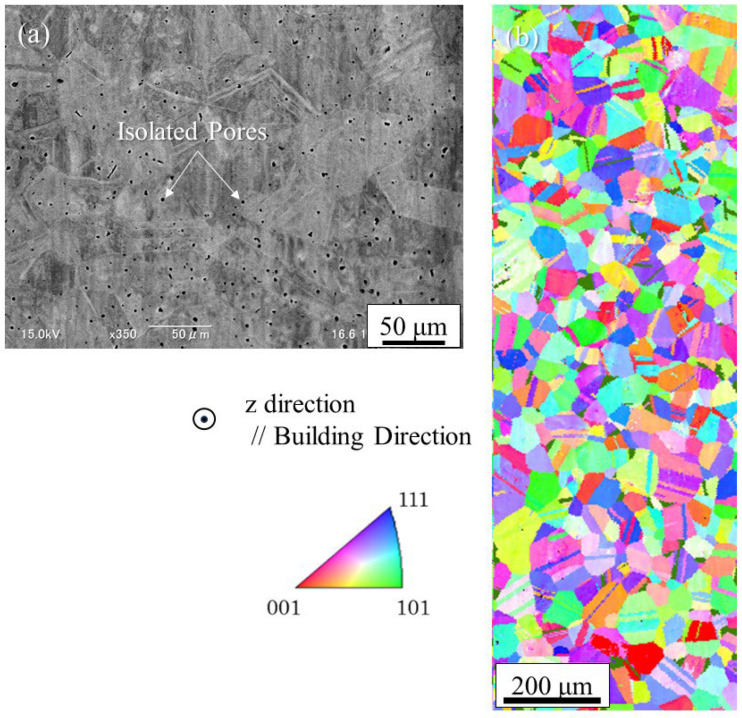
The microstructures of the SUS316L sample manufactured using Studio System^TM^ technology: (**a**) SEM image and (**b**) IPF map.

Figure 25 shows the microstructures of the SUS316L sample manufactured using Studio System^TM^ technology. Figure 25a shows the SEM microstructure of the SUS316L sample manufactured by the MEX method. Many black parts, indicated by arrows, are visible. Most of the black parts seen in the SEM microstructure are isolated pores formed during the sintering process, which are also formed by the MIM technique. In principle, these pores cannot be eliminated. However, unlike blowholes formed by casting or defects formed by the PBF and DED methods, the microscopic cavities are dispersed throughout, as shown in Figure 25a. Therefore, it is believed that they will not cause accidental or localized reductions in mechanical properties or density, but instead result in a homogeneous structure. The IPF map obtained by the EBSD observation is shown in Figure 25b. As can be seen, there are grains smaller than 100 μm without texture. The grain size and aspect ratio of the sample were evaluated for a total number of 248 grains, and the average grain size and aspect ratio were found to be 24.3 ± 7.9 μm and 1.61 ± 0.7, respectively. It can also be seen that many of the grains have twins. Therefore, the SUS316L sample produced by the sintering method is characterized as having a random orientation microstructure.

### 5.2. SUS316L Samples Manufactured by DM P2500

**Figure 26:**
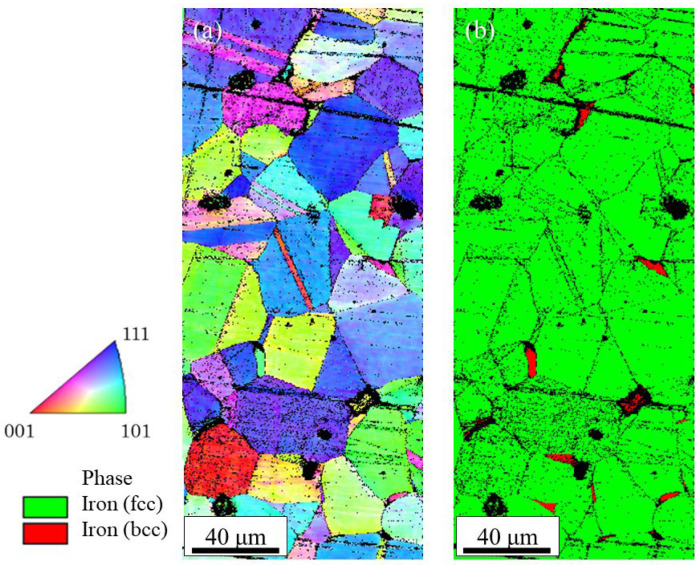
The microstructures of the SUS316L sample manufactured using the DM P2500: (**a**) IPF map and (**b**) phase map.

The SUS316L sample was also manufactured using the BJT method using DM P2500 (Markforged, Waltham, MA, USA) [55,56]. Figure 26a and Figure 26b show the IPF map and the phase map of the sample, obtained by EBSD observation, respectively. As can be seen in Figure 26a, random grains can be observed with some twins. The crystal structure of some grains, especially smaller grains, is not fcc but bcc, as shown in Figure 26b. As a result, the manufactured part shows weak ferromagnetism. It can be concluded that SUS316L samples without texture can be manufactured using the sintering method, including the MEX and BJT methods.

### 5.3. Difference in Microstructures Manufactured by DED and MEX/BJT

The SUS316L powder for AM is usually produced by the gas atomization method, where the cooling rate in the gas atomization of the molten metal is very high [57]. Although SUS316L is an austenitic stainless steel, some particles have a bcc or bct structure, as shown in Figure 7. This means that these particles with the bcc or bct structures are ferrite or martensite. Here, considering the phase of these particles, the phase of these particles would be ferrite. Usually, in the case of martensite, the half-width of the peaks obtained by XRD are large due to the large strain inside the martensite. However, as seen in Figure 7, the half-widths of these bcc or bct peaks are small. Furthermore, martensitic transformation occurs at the start temperature of the martensitic transformation, regardless of the cooling rate. In a previous study, it has been reported that the welded SUS316L (AISI316L) often contains δ-ferrite in an austenite matrix because the Mo in the SUS316L stabilizes the primary δ-ferrite [58]. Because of this, the phase of these particles would be δ-ferrite.

In AM using MEX and BJT methods, since the samples are produced using powder metallurgy without the melting and solidification process, the structure of the raw powder is directly reflected in the structure of the manufactured sample. Therefore, although the manufactured sample has random crystal orientation, some grains have a bcc structure. In contrast, in the DED method, even though SUS316L powder with a partially bcc structure was used as the raw powder, the bcc structure did not remain in the resulting product. This is because DED occurs in the melting and solidification process. However, columnar crystallization and texture formation occur as a result of solidification under a strong temperature gradient. These drawbacks can be overcome by the addition of heterogeneous nucleation site particles.

The mechanism of the effect of SrO on the rapid solidification process of DED can be understood by the heterogeneous nucleation theory. The presence of SrO heterogeneous nucleation site particles lowers the interfacial energy barrier, and the lowered free energy barrier for nucleation leads to a decrease in the supercooling needed to initiate the nucleation events, which promotes the formation of equiaxed grains rather than columnar grains. Furthermore, the dispersed nucleation induced by SrO heterogeneous nucleation site particles stabilizes the solidification front under a sharp temperature gradient, suppressing the formation of defects. These results clarify the role of the SrO heterogeneous nucleation site particles in improving the microstructural homogeneity and formability of SUS316L produced by the DED method.

In this study, it is found that the effects of the addition of SrO heterogeneous nucleation site particles on the microstructure and formability of SUS316L samples manufactured with ALPION Series machine are different from those with LAMDA 200 machine. This is thought to be due to differences in the melting state, including temperature, cooling conditions, and re-heating by layering. The volumetric energy density of LAMDA (60 to 150 J/mm^3^) is smaller than that of ALPION (500 J/mm^3^). This is because the hatch spacing and layer thickness for the fine-laser cladding system of ALPION are small, so the calculated volumetric energy density results in a large value. Therefore, the abovementioned phenomenon cannot be explained by the difference in the size of the volumetric energy density. The residence time in the liquid state of the melt pool is the most important parameter that controls the chemical homogeneity of the samples. The residence time can be estimated approximately by dividing the maximum extent of the melt pool along the direction of laser travel by the laser scanning speed [59]. The former and later values for the LAMDA 200 machine and ALPION samples are 3.5 mm, 13.3 mm/s, and 1 mm, 10 mm/s, respectively. Therefore, the residence time for LAMDA samples is estimated to be 263 ms and 100 ms for ALPION samples. These values are larger than that of the PBF sample, especially in the case of the LAMDA sample. The longer residence time causes the decomposition of heterogeneous nucleation site particles, resulting in a loss of grain-refining efficiency. This phenomenon is known as the “fading effect” in casting [60]. This is why the grain refinement observed in the ALPION sample was not found in the LAMDA sample. However, these results are based on limited samples manufactured under limited conditions and do not apply universally. We are now studying new heterogeneous nucleation sites that work universally on all DED machines, which will be presented in the future. Since the results obtained were from the use of powder-type DED machines and SUS316L stainless steel of which there are other types, similar effects can be expected for other materials and wire-type DED machines. Therefore, the technique can be applied not only to AM but also to welding.

## 6. Conclusions

This study investigates the manufacturing of SUS316L samples using two different directed energy deposition (DED) machines, the ALPION Series machine and the LAMDA 200 machine, with and without the addition of SrO heterogeneous nucleation site particles. The ALPION Series machine is a multi-beam type while the LAMDA 200 machine is a single-beam type. As a result, it is found that the effects of the addition of SrO heterogeneous nucleation site particles on the microstructure and formability of the sample are different for these machines. The details of the results obtained are summarized as follows:

(1) When using the ALPION Series machine, unidirectional and cross-scanning strategies were employed. The inoculated samples with SrO particles demonstrated fewer defects and had refined, equiaxed grain structures compared to those of the uninoculated samples. This indicates that SrO particles effectively reduce defects and refine microstructure in the powder-based DED method.
(2) When using the LAMDA 200 machine, the SUS316L samples were assessed for surface defects, which were found to be reduced after trimming. The study concluded that the addition of SrO particles not only improves the formability of the samples but also significantly reduces the defect volume and suppresses texture formation. Although SrO inoculation did not cause grain refinement as a result of high-temperature conditions, it improved randomness in crystal orientation.
(3) The effects of the addition of SrO heterogeneous nucleation site particles on the microstructure and formability of SUS316L samples manufactured using the ALPION Series machine and LAMDA 200 machine are different. This is thought to be due to differences in the melting state, including temperature, cooling conditions, and reheating. In the future, we would like to study new heterogeneous nucleation sites that work universally on all DED machines.

## Figures and Tables

**Table 1:** The chemical compositions of the larger and smaller SUS316L powders (mass%).

	Ni	Cr	Mo	Mn	C	Si	P	S	Fe
Larger SUS316L	12.58	17.40	2.06	0.67	0.010	0.91	0.005	0.003	Bal.
Smaller SUS316L	12.58	16.51	2.04	0.24	0.015	0.80	0.017	0.006	Bal.

**Table 2:** The process parameters for the LAMDA 200 machine and the ALPION Series machine.

Machine	Sample Size, *l* mm × *w* mm × *h* mm	Number of Layer, *n*	Laser Power, *P*/W	Scanning Speed, *v*/mm·s^−1^	Hatch Spacing, *s*/mm	Layer Thickness, *t*/mm	Scanning Strategy
LAMDA	10 × 10 × 10	20	400, 600, 800, 1000	13.3	1	0.5	Zigzag
ALPION	15 × 1 × 10	68	150	10	0.2	0.15	Cross-scanning, Unidirectional

**Table 3:** Average grain size and aspect ratio calculated using EBSD measurement data for samples manufactured by the ALPION Series machine. The standard deviation (*SD*) and the number of grains analyzed (*N*) are also shown in this table.

	Uninoculated Sample	Inoculated Sample	
		Elongated Grain	Fine Grain
Average Length of Longer Axis, *D_L_*/μm	252.4 ± 130.9 (*N* = 34)	178.8 ± 95.7 (*N* = 40)	34.8 ± 25.2 (*N* = 26)
Average Length of Shorter Axis, *D_S_*/μm	35.8 ± 16.1 (*N* = 34)	29.4 ± 8.5 (*N* = 40)	14.1 ± 6.8 (*N* = 26)
Aspect Ratio, *AR*	7.58 ± 3.75 (*N* = 34)	6.53 ± 3.57 (*N* = 40)	2.54 ± 1.15 (*N* = 26)
Average Size, *D_ave_*/μm	119.5 ± 86.5 (*N* = 44)	41.4 ± 25.9 (*N* = 22)	

**Table 4:** Average grain size and aspect ratio calculated using EBSD measurement data for samples manufactured by the LAMDA 200 machine. The standard deviation (*SD*) and the number of grains analyzed (*N*) are also shown in this table.

		Uninoculated Sample	Inoculated Sample
x-z plane	Average Length of Longer Axis, *D_L_*/μm	229.2 ± 89.8 (*N* = 51)	285.4 ± 107.3 (*N* = 65)
	Average Length of Shorter Axis, *D_S_*/μm	52.9 ± 22.5 (*N* = 51)	59.4 ± 21.4 (*N* = 65)
	Aspect Ratio, *AR*	4.66 ± 23.8 (*N* = 51)	5.44 ± 18.3 (*N* = 65)
	Average Size, *D_ave_*/μm	221.3 ± 89.2 (*N* = 51)	262.1 ± 89.2 (*N* = 65)
y-z plane	Average Length of Longer Axis, *D_L_*/μm	282.4 ± 57.6 (*N* = 81)	400.9 ± 110.4 (*N* = 55)
	Average Length of Shorter Axis, *D_S_*/μm	57.6 ± 20.2 (*N* = 81)	108.9 ± 29.9 (*N* = 55)
	Aspect Ratio, *AR*	4.88 ± 15.9 (*N* = 81)	3.95 ± 32.8 (*N* = 55)
	Average Size, *D_ave_*/μm	246.2 ± 80.0 (*N* = 81)	404.5 ± 109.1 (*N* = 55)

## Data Availability

The original contributions presented in this study are included in the article. Further inquiries can be directed to the corresponding author.
